# Prediction and Analysis of Ultimate Bearing Capacity of Square CFST Long Column under Eccentric Compression after Acid Rain Corrosion

**DOI:** 10.3390/ma14102568

**Published:** 2021-05-14

**Authors:** Xuetao Lyu, Liqiang Zhang, Tong Zhang, Ben Li, Huan Li, Yang Yu

**Affiliations:** 1 Shaanxi Key Laboratory of Safety and Durability of Concrete Structures, Xijing University, Xi’an 710123, China; 2 Advanced and Sustainable Infrastructure Materials Group, School of Transportation, Civil Engineering and Architecture, Foshan University, Foshan 528000, China; zlq0227@126.com; 3 School of Civil Engineering, Liaoning Technical University, Fuxin 123000, China; sktm1@163.com (L.Z.); zt_1987_zt@163.com (T.Z.); 4 School of Civil and Environmental Engineering, University of Technology Sydney, Ultimo, NSW 2007, Australia; 5 Centre for Infrastructure Engineering, Western Sydney University, Penrith, NSW 2751, Australia

**Keywords:** acid rain corrosion, concrete-filled square steel tube long column, eccentric compression, ultimate bearing capacity, finite element analysis

## Abstract

This paper adopts the method of steel tube wall thickness and strength reduction to simulate corrosion damage. The numerical model of the square concrete-filled steel tube long column (SCFST-LC) under eccentric compression after acid rain corrosion is established in the finite element software, ABAQUS. The reliability and accuracy of the model are verified by comparing it with published relevant experimental results. The failure mode, load-deformation curve, and ultimate compressive load were analysed. Following that, the impacts of section size, yield strength of the steel tube, axial compressive strength of concrete, steel ratio, slenderness ratio, and load eccentricity on its ultimate compressive load are comprehensively investigated. The results demonstrate that the ultimate compressive load of the SCFST-LC decreases significantly with the increase in corrosion rate. The corrosion rate increases from 10 to 40%, and the ultimate bearing capacity decreases by 37.6%. Its ultimate bearing capacity can be enhanced due to the increase in section size, material strength, and steel ratio. In contrast, the ascending slenderness ratio and load eccentricity has harmful effects on the ultimate compressive load of the specimens. Finally, a simplified formula for the axial compressive load of the SCFST-LC under eccentric compression after acid rain corrosion is proposed. The calculation accuracy is high and the deviation of the results is basically within 15%, which is in good agreement with the numerical simulation results.

## 1. Introduction

The concrete-filled steel tube column [1,2,3], as a composite structure, is formed by filling a steel tube with concrete, in which the external steel tube and internal core concrete bear load together. Compared with ordinary concrete columns, because the external steel tube of concrete filled steel tube (CFST) has a restraining effect on the lateral expansion of core concrete, CFST columns have more advantages in strength and ductility than ordinary concrete columns [4,5]. Due to its desirable mechanical properties and economic and construction benefits, concrete-filled steel tube columns have been widely implemented in both mechanical and civil engineering fields, such as combined bridges, super high-rise buildings, subway stations, and industrial plants [6,7,8]. However, with the deterioration of the global environment, they unavoidably undergo the impacts of climatic conditions and human factors during their service, e.g., acid rain, low temperature, etc. According to meteorological data, acid rain pollution in China has been gradually expanding from south to north and has become more and more serious in most cities since 2000 [9,10]. When the concrete-filled steel tube columns are exposed to an acid rain environment for a long time, the external steel tubes will be subjected to severe surface corrosion, which can result in a significant reduction in their mechanical properties [11]. Those adverse environmental conditions will greatly threaten the safety and durability of concrete-filled steel tube columns.

Therefore, more and more scholars have carried out research on concrete-filled steel tube columns under environmental corrosion [12,13,14,15,16,17]. Li et al. [18] established the finite element model of a double-layer concrete-filled steel tube short column and studied its mechanical properties under continuous load and chloride ion corrosion. The numerical results showed that the bearing capacity of the concrete-filled steel tube short column was significantly reduced after chloride ion corrosion. Han et al. [19,20,21,22,23] experimentally investigated and theoretically analysed the performance degradation of 17 concrete-filled square steel tube columns, which worked under long-term load and chloride ion corrosion. Three types of corrosion conditions were taken into consideration, including non-corrosion, semi-immersion corrosion, and full immersion corrosion. The compression, tension, and bending of the concrete-filled square steel tube columns were analysed. Additionally, a simplified calculation formula for the bearing capacity of the concrete-filled steel tube column after chloride ion corrosion was proposed. Zhang et al. [24] carried out an axial static load test on 11 thin-walled circular concrete-filled steel tube short columns in a corrosive environment. The experimental results demonstrated that the failure mode of those columns with chloride corrosion was typical partial outward bulging shear failure, which is different from those without corrosion. The ultimate bearing capacity and plastic deformation capacity of those concrete columns decreased significantly with the corrosion rate increasing. Gao et al. [17] conducted axial compression tests on 18 concrete-filled steel tube short columns after being corroded by salt spray. The results showed that the salt spray corrosion would diminish the steel ratio and change the mechanical properties of steel tubes. Chen et al. [16,25,26,27,28,29] conducted experimental research and theoretical investigations on the concrete-filled steel tube short columns under acid rain corrosion in Jiangxi Province, China. Based on the results, a simplified calculation formula for the bearing capacity of a concrete-filled steel tube short column, corroded by acid rain, was proposed. The calculation method of its flexural bearing capacity for seismic design was also given.

At present, most of the published papers on concrete-filled steel tube columns under environmental corrosion are based on short columns under axial compression. According to the slenderness ratio of CFST members, a column with a slenderness ratio greater than 4 is a long column, and a column with a slenderness ratio less than 4 is a short column. There is very little existing research concerning the SCFST-LC under eccentric compression after acid rain corrosion. Therefore, this paper takes the SCFST-LC under eccentric compression, corroded by acid rain, as the research object. By modifying the yield strength and wall thickness of the external steel tube, its finite element model is established. Based on a representative numerical example, its whole eccentric compression process is numerically investigated. The mechanical mechanism and mechanical properties of the SCFST-LC under eccentric compression after acid rain corrosion are comprehensively analysed. Moreover, a parametric analysis is conducted to investigate the importance and effects of its main parameters on the ultimate bearing capacity of the SCFST-LC, under eccentric compression after acid rain corrosion.

## 2. Finite Element Model of the CFST-LC under Eccentric Compression

### 2.1. Stress-Strain Relationship Model

The CFST-LC is composed of two materials, i.e., steel and concrete. Due to the influence of acid rain, not only the effective thickness of the external steel pipe is reduced, but its mechanical properties are also weakened. According to the existing research results, it is assumed that the surface of the steel pipe is uniformly corroded, and the thickness of the steel pipe is uniformly reduced. Therefore, the stress-strain relationship model of steel adopted in this paper is based on the model proposed in [29], which considers the influence of the corrosion damage on the external steel pipe. The concrete stress-strain relationship model improved by Liu [30] is suitable for calculating the core concrete in the steel tube and can be utilized to describe the constitutive relationship of concrete under eccentric compression. The specific expressions are as follows:

1. Stress-strain relationship model of steel
  (1) $$\sigma_{s}=\left\{\begin{array}{c} E_{se}\varepsilon_{s},\;\;\;\;\;\;\varepsilon_{s}<\varepsilon_{e}\\ -A\varepsilon_{s}^{2}+B\varepsilon_{s}+C,\;\varepsilon_{e}<\varepsilon_{s}\leq \varepsilon_{e1}\\ f_{ye,\;\;\;\;\;\;\;\;\;\;\;\;}\varepsilon_{e1}<\varepsilon_{s}\leq \varepsilon_{e2}\\ f_{ye}\left[1+0.6\frac{\varepsilon_{s}-\varepsilon_{e2}}{\varepsilon_{e3}-\varepsilon_{e2}}\right],\;\varepsilon_{e2}<\varepsilon_{s}\leq \varepsilon_{e3}\\ 1.6f_{ye},\;\;\;\;\;\;\varepsilon_{s}>\varepsilon_{e3} \end{array}\right.$$
  (2) $$\varepsilon =0.8f_{ye}/E_{se}$$
  (3) $$\varepsilon_{e1}=1.5\varepsilon_{e}$$
  (4) $$\varepsilon_{e2}=10\varepsilon_{e1}$$
  (5) $$\varepsilon_{e3}=100\varepsilon_{1}$$
  (6) $$A=0.2f_{y}{\left(\varepsilon_{e1}-\varepsilon_{e}\right)}^{2}$$
  (7) $$B=2A\varepsilon_{e1}$$
  (8) $$C=0.8f_{ye}+A\varepsilon_{e}{}^{2}-B\varepsilon_{e}$$
  (9) $$E_{se}=(1-0.955\gamma )E_{e}$$
  (10) $$f_{ye}=(1-1.007\gamma )f_{y}$$
  (11) γ=Δt/t
  (12) $$\Delta t=t-t_{e}$$
  where *E_s_* is the initial elastic modulus of the steel, MPa; *E_se_* is the effective elastic modulus of the steel after corrosion, MPa; *f_y_* is the initial yield strength of the steel, MPa; *f_ye_* is the effective yield strength of the steel after corrosion, MPa; *γ* is the corrosion rate measured by the test, %; ∆*t* is the reduction of the wall thickness of the steel tube due to corrosion, mm; *t* is the initial wall thickness of the steel tube, mm; *t_e_* is the effective wall thickness of the steel tube after corrosion, mm.
2. Stress-strain relationship model of concrete
  (13) $$y=\left\{\begin{array}{l} 2x-x^{2}\;\;\;\;(x\leq 1)\\ \frac{x}{\beta {\left(x-1\right)}^{\eta }+x}\;\;\;(x>1) \end{array}\right.$$
  (14) $$x=\varepsilon /\varepsilon_{0}$$
  (15) $$y=\sigma /\sigma_{0}$$
  (16) $$\sigma_{0}=f_{c}^{\prime }$$
  (17) $$\varepsilon_{0}=(1300+12.5f_{c}^{\prime }+800\zeta_{e}^{0.2})\times 10^{-6}$$
  (18) η=1.6+1.5/x
  (19) $$\beta ={\left(f_{c}^{\prime }\right)}^{0.1}/\left[1.2\sqrt{1+\zeta_{e}}\right]$$
  (20) $$f_{c}^{\prime }=\left[0.76+0.2+\log_{10}\left(\frac{f_{cu}}{19.6}\right)\right]f_{cu}$$
  (21) $$\zeta_{e}=\alpha_{e}\times f_{ye}/f_{ck}$$
  (22) $$f_{ck}=0.88\times 0.76\times f_{cu}$$
  (23) $$\alpha_{e}=A_{se}/A_{c}$$
  where, *σ*_0_ and *ε*_0_ are the peak stress and peak strain of concrete core, respectively; *f_c_′* is the compressive strength of the concrete cylinder, MPa; *f_cu_* is the compressive strength of the concrete cube, MPa; *f_ck_* is the standard value of axial compressive strength of the concrete, MPa; *ξ_e_* is the effective restraint effect coefficient of the concrete-filled steel tube; *α_e_* is the effective steel ratio of the steel tube; *A_se_* is the effective cross-section area of the steel tube, mm^2^; *A_c_* is the cross-section area of the concrete column, mm^2^.

The Poisson’s ratio (*ν*) of the concrete is set to 0.2 [31]. The values of other parameters of the concrete, including its elastic modulus (*E_c_*), dilation angle (Ψ), and ratio of the second stress invariant on the tension-compression meridian plane (*K_c_*), as well as *f_b_*_0_/*f_c_*_0_ ratio, can refer to [31]. The formulas are given by Equations (24)–(27). The eccentricity in-flow rule is set to 0.1. To improve the convergence, the viscosity parameter (*μ*) is 0.0005.

(24) $$E_{c}=4700\sqrt{f_{c}^{\prime }}$$

(25) $$\Psi =\left[\begin{array}{l} 56.3(1-\zeta_{e})\\ 6.672e^{\frac{7.4}{4.64+\zeta_{e}}} \end{array}\right]\begin{array}{l} for\;\zeta_{e}\leq 0.5\\ for\;\zeta_{e}>0.5 \end{array}$$

(26) $$K_{c}=\frac{5.5}{5+2{\left(f_{c}^{\prime }\right)}^{0.075}}$$

(27) $$f_{b0}/f_{c0}=1.5{\left(f_{c}^{\prime }\right)}^{-0.075}$$

### 2.2. Modeling Statements and Parameter Definitions

**Figure 1:**
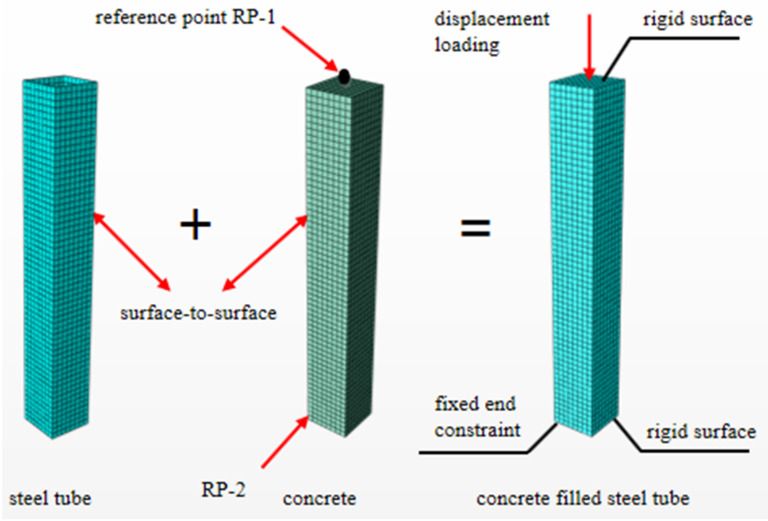
Schematic diagram of mesh dividing and boundary conditions of the finite element model.

The numerical modelling and calculation is conducted in finite element software ABAQUS (v.6.14). In the simulation, ABAQUS plastic analysis model was used for the constitutive analysis of steel, and the plastic damage model in the calculation software is used for the concrete constitutive model. The steel tube and concrete are established by the four-node, three-dimensional shell element, S4R, and the eight-node, three-dimensional solid element, C3D8R, respectively. The mesh of the steel tube is set to a quadrilateral element and that for the concrete is determined as a hexahedral element. To improve the accuracy and efficiency of the calculation, the mesh size is taken as 1/10 of the section length of the model [32]. To guarantee the calculation accuracy in the thickness direction of the shell element, the Simpson integration method, with 9 integration points, is utilised. In the construction application of concrete-filled steel tubular members, there are more or less initial bending, and the section is not completely symmetrical, thus forming the initial eccentricity. In order to better meet the actual situation, the initial defects of the components are established in the calculation. Therefore, the initial defect is taken as 1/1000 of the length of the component, with consideration to its geometric nonlinearity [1]. The initial defect is taken as 1/1000 of the length of the component with consideration to its geometric nonlinearity. The contact between the steel tube and concrete adopts surface-to-surface, of which the normal direction is hard contact. The outer surface of the concrete is a master surface, and the inner surface of the steel tube is a slave surface. The tangential friction force (Friction Coeff) is taken as 0.6 [33]. For the boundary conditions of this model, its one end is set as a consolidation, that is to constrain all 6 degrees of freedom. Another end adopts the displacement loading mode, which means a vertical displacement is applied in the Z direction, while the other 5 degrees of freedom are constrained. To improve the calculation convergence and avoid stress concentration, both ends of the model are set as rigid surfaces (rigid body). The geometric centre point of each end surface is selected as reference points (reference point), i.e., RP-1 and RP-2. The region type (region type) is defined as the corresponding end face of each reference point. The mesh dividing and boundary conditions of the finite element model are shown in Figure 1.

### 2.3. Model Verification

**Figure 2:**
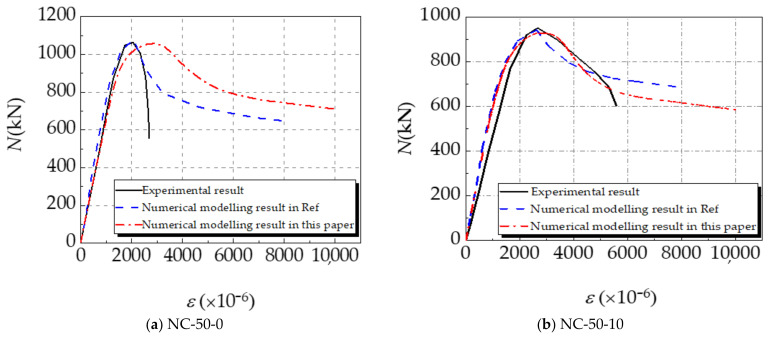

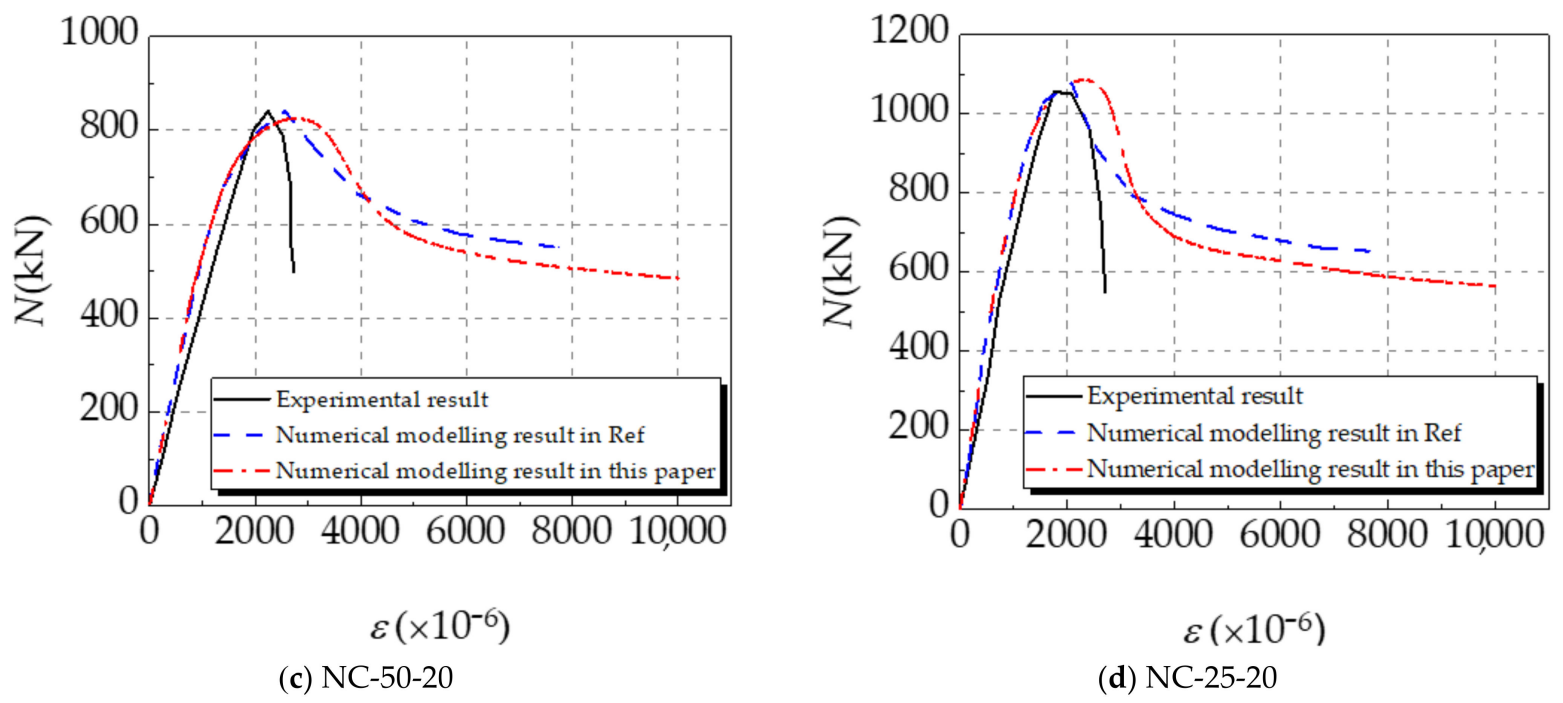
Simulation verification results of Ref. [34]. (**a**) NC-50-0; (**b**) NC-50-10; (**c**) NC-50-20; (**d**) NC-25-20.

**Figure 3:**
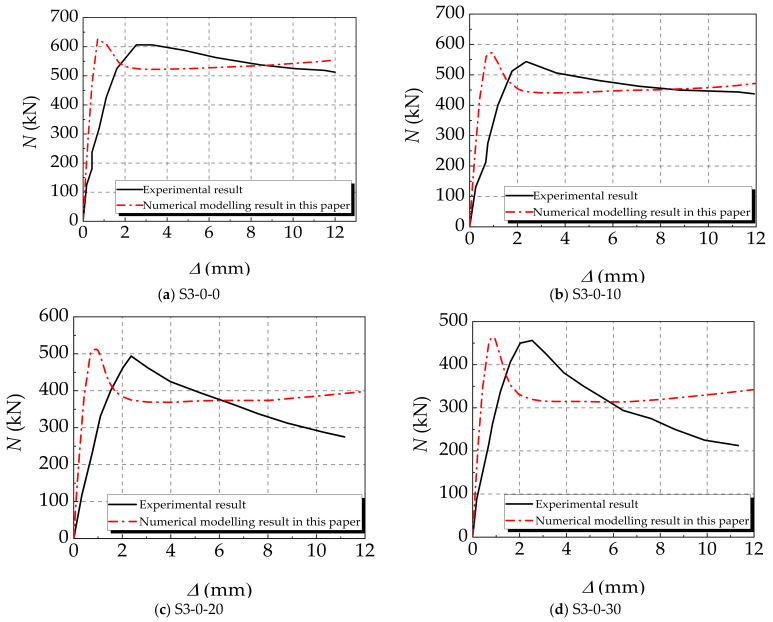

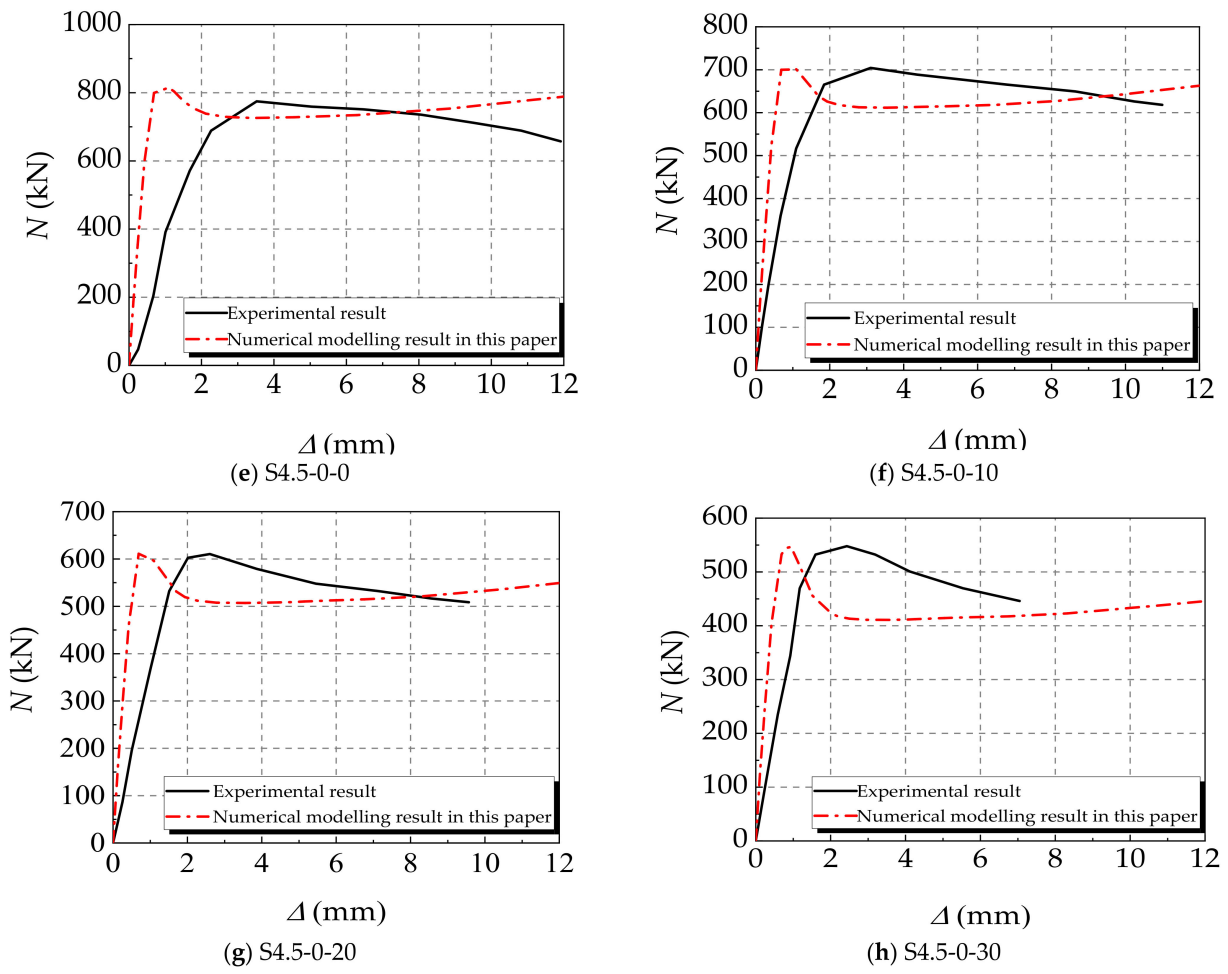
Results verification of Ref. [35]. (**a**) S3-0-0; (**b**) S3-0-10; (**c**) S3-0-20; (**d**) S3-0-30; (**e**) S4.5-0-0; (**f**) S4.5-0-10; (**g**) S4.5-0-20; (**h**) S4.5-0-30.

The concrete-filled square steel tube long columns under eccentric compression in [34] and the concrete-filled square steel tube short columns under axial compression in [35] are simulated and verified with the established numerical model. The parameters of specimens in [34,35] are given in Table 1. The numerical modelling results of the ultimate bearing capacity of each specimen are obtained and shown in Table 2. According to the results, due to the difference in the constitutive selection of corroded steel and concrete in the literature and the finite element simulation in this paper, the corresponding calculation results are different, which leads to the higher simulation results in the literature than in this paper. The values of *N_ue_*/*N_be_* of specimens are all within 10% and the average value of *N_ue_*/*N_be_* is approximately equal to the average value of *N_ue_*/*N_ce_*, which proves the rationality of the established model. The relationship curves of the eccentric compression load *N* vs. vertical strain *ε* (ratio of vertical displacement increment to member length) and the axial compression load *N* vs. axial displacement Δ (axial compression displacement increment) are depicted in Figure 2 and Figure 3. Those curves demonstrate that the trend of the numerical modelling results coincide with the experimental results in [34,35]. However, there are differences between the initial stiffness value and the ultimate bearing capacity of the specimen. The initial stiffness value of the simulation curve in the figure is slightly higher than that of the test curve. There is a gap between the high and low ultimate bearing capacity in Figure 2. This is mainly because the corrosion of the component is locally uneven during the corrosion test, and there will be different degrees of initial defects before the test. The finite element modelling assumes that all materials are in an ideal state, which leads to the difference between the experimental and simulated ultimate bearing capacity of corroded and non-corroded members. Besides, the experimental measurement errors of axial displacement can also result in the difference between experimental and numerical results. According to the descending branches of those curves, except for specimen NC-50-0, the results calculated by the proposed numerical model are more consistent with the experimental results than the numerical modelling results in [34,35]. It further verifies the feasibility and reliability of the model proposed in this paper.

## 3. Results and Discussion of CFST-LC under Eccentric Compression

### 3.1. Numerical Model under Eccentric Compression

To study the influence of acid rain corrosion on the load-bearing capacity of the concrete-filled steel tube long column under eccentric compression, a set of finite element models are designed. The design parameters of these models are shown in Table 3. Poisson ratios of concrete *μ*_c_ and steel *μ*_s_ are 0.2 and 0.3, respectively.

### 3.2. Failure Mode

**Figure 4:**
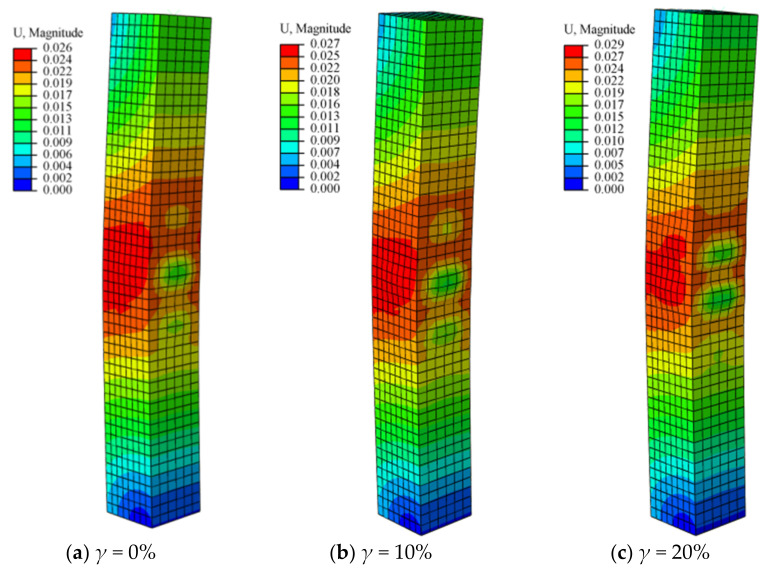

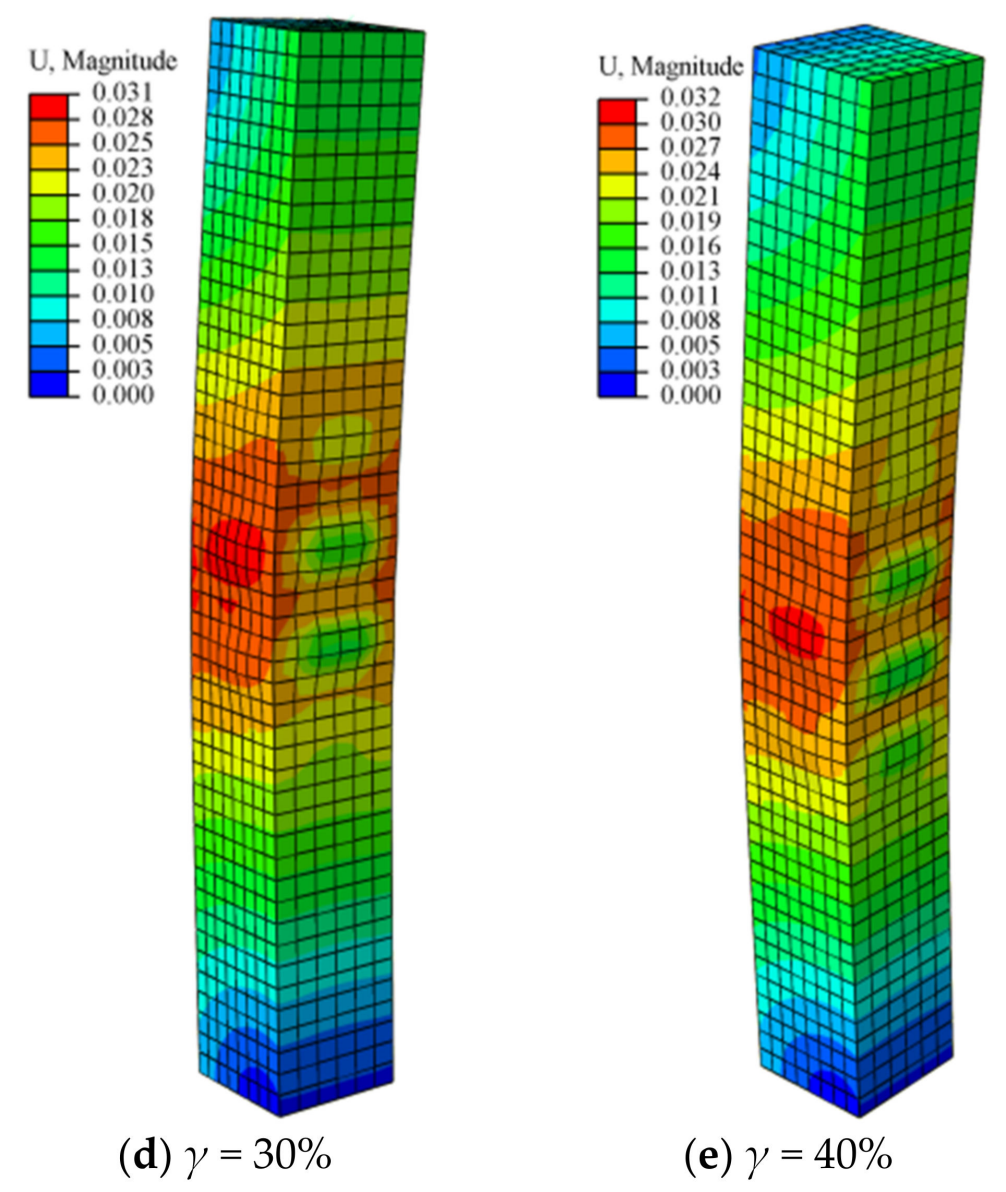
Schematic diagram of the failure mode of concrete-filled square steel tube long column subjected to eccentric loads with different corrosion rates (**a**) *γ* = 0%; (**b**) *γ* = 10%; (**c**) *γ* = 20%; (**d**) *γ* = 30%; (**e**) *γ* = 40% (U is the buckling displacement, unit: mm).

The schematic diagram of the failure mode of the CFST-LC, subjected to eccentric loads after undergoing different degrees of acid rain corrosion, is shown in Figure 4. The failure modes of the CFST-LC under eccentric compression with corrosion (in Figure 4b–e) are similar to those without corrosion plotted in Figure 4a. Both of them have local buckling occurring in the middle of the long column, where the mid-span deflection is the largest. In practice, due to the defects in steel tubes, local buckling can take place at any position along the column height. The buckling directions of those CFST-LC in all faces are outside because the internal core concrete provides the external steel tube with effective support.

With the corrosion rate rising, the mid-span deflection of the specimen is largely amplified, increasing from 26 mm under *γ* = 0% to 32 mm under *γ* = 40%. It can be noted that the effects of the corrosion rate on the magnitude of mid-span deflection during the range that *γ* = 0% to *γ* = 30% is more significant than that under *γ* = 30% to *γ* = 40%. Since the specimens utilized in this paper are uniformly corroded, the corrosion degrees of steel tubes are approximately the same. The following prerequisite indicated that if the defect in steel is almost the same, then the middle of the (long column) LC becomes the weakest district. With the corrosion rate increasing gradually, the possibility of local defection on the surface of the steel tube increases where the degree of local buckling becomes greater. However, when the steel tube is seriously corroded, its effective thickness will be largely reduced. Hence, the specimen will fail in advance, resulting in small deformation.

**Figure 5:**
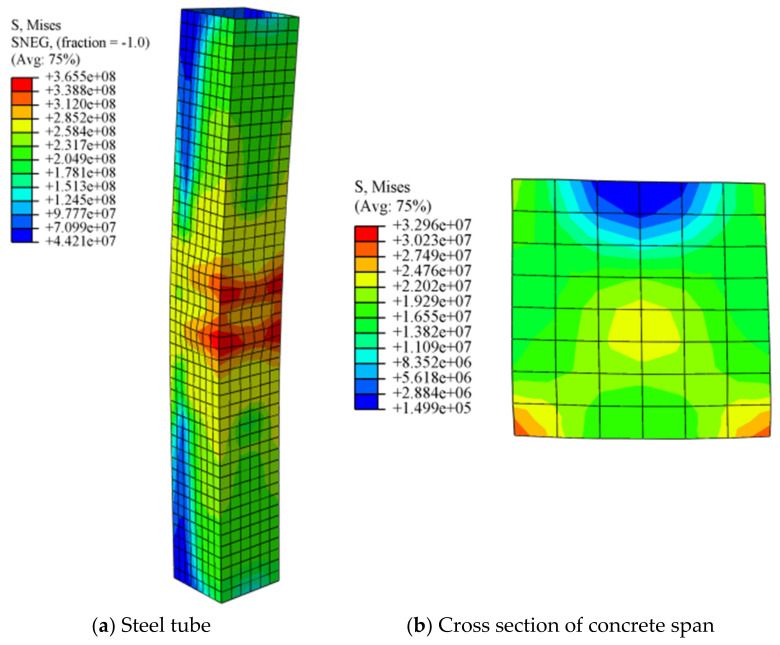
Stress cloud diagram (**a**) steel tube (**b**) concrete.

When the specimen S-40-345-0.11-20 fails, the stress cloud diagrams of its steel tube and concrete are shown in Figure 5. When the specimen is uniformly corroded and subjected to an eccentric load, the stress distribution in each direction is different, causing it to become concentrated in the buckling part of the steel tube and the failure part of the concrete. According to Figure 5a, along the height of steel tube, the maximum stress appears in the mid-span section, where the stress at the corner reaches the maximum value of 365 MPa. It is caused by stress concentration, which indicates that the restraint effect of the concrete is more effective at the corner position. The stress mitigates gradually from the middle section to the two ends along the column height. The stress differences between the compression side and the tension side, as well as the mid-span section and end section of the steel tube, are considerably different. This is because the LC has rotated due to the eccentric compression and the inconsistency of the range, leading to the different constraints at the ends of the column. In Figure 5b, as the stress cloud diagram of the mid-span cross-section area of the concrete depicts, the maximum stress appears at the two compression corners of the cross-section area. It is about 32.96 MPa, which demonstrates that the constraining effect of the steel tube is the most significant in this position. Along the direction of the compressive edge to the tensile edge of the concrete cross section, the stress decreases continuously, and the minimum value is 19 MPa. By comparing the stress cloud diagrams of the steel tube and core concrete, most of the vertical load of the concrete-filled square steel tube is supported by the core concrete during the entire eccentric compression process. When the eccentric load applied on the entire specimen exceeds the threshold, the core concrete reaches its ultimate load and the steel tube yields.

**Figure 6:**
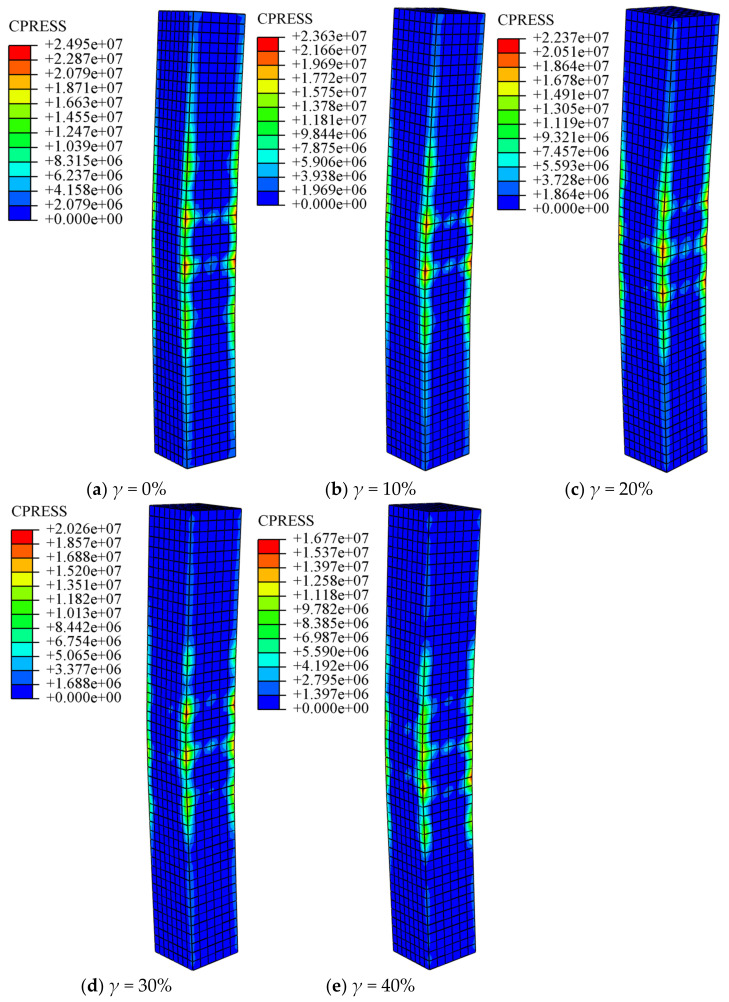
Steel tube-concrete normal contact force distribution at peak load point (unit: N). (**a**) *γ* = 0%; (**b**) *γ* = 10%; (**c**) *γ* = 20%; (**d**) *γ* = 30%; (**e**) *γ* = 40%.

Figure 6 shows the distribution of normal contact force between steel tube and concrete when the specimen reaches the peak load. It can be seen that the distribution of normal contact force between steel tube and core concrete is mainly concentrated in the corner, and the steel tube has less binding force on concrete at the end of the member. It can be seen from the figure that with the increase in the degree of corrosion, the binding force of the steel tube on core concrete becomes smaller and smaller, which is mainly due to the reduction in the thickness of the steel tube wall, caused by corrosion, thus reducing the effect of the steel tube on concrete. With this increase in the degree of corrosion, the bending failure characteristics of specimens become more obvious.

### 3.3. Load-Vertical Displacement Relationships

**Figure 7:**
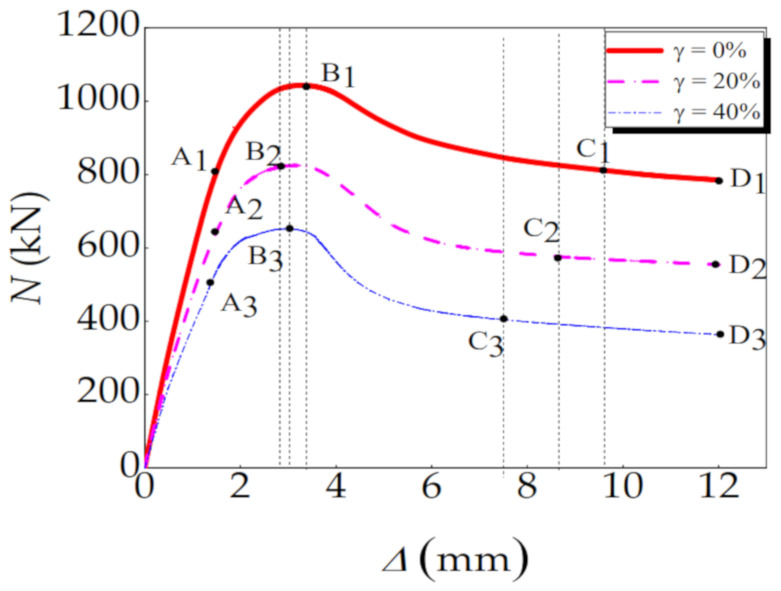
Load-vertical displacement curve of specimen.

Figure 7 shows the load-vertical displacement relationship curves of a typical CFST-LC under eccentric compression after acid rain corrosion. The trends of these curves are basically the same under different corrosion rates. The loading process can be roughly divided into 4 stages: linear stage (0A_i_), non-linear stage (A_i_B_i_), descending stage (B_i_C_i_), and smooth phase (C_i_D_i_). The characteristics of each stage are analysed as follows:

1. Within the linear stage (0A_i_), the relationship between load and vertical displacement is approximately linear. The external steel tube and internal concrete work independently, the interaction force between which is 0. Compared with the specimen that has not been corroded, displayed in the red line, the specimens undergoing acid rain corrosion will enter the elastic-plastic phase earlier. Additionally, with the corrosion rate rising, their elastic modulus diminish significantly, and elastic stages are slightly shortened.
2. During the non-linear stage (A_i_B_i_), with the increase in applied load, small cracks appear in the core concrete and develop gradually. Meanwhile, since the transverse deformation coefficient of the core concrete is larger than that of the steel pipe, the steel pipe will exert a lateral restraint on the core concrete. The specimen reaches its ultimate bearing capacity at B_i_ point. Compared with the specimens without corrosion, the specimens will reach the ultimate bearing capacity ahead of time, and with the increase in corrosion rate, the ultimate bearing capacity of the specimens gradually decreases, and the time to reach the ultimate bearing capacity is also earlier and earlier.
3. Within the decline stage (B_i_C_i_), after the specimen reaches its ultimate bearing capacity, the core concrete is crushed, and the steel tube yields. Hence, the specimen undergoes plastic failure. Furthermore, the acid rain corrosion can largely shorten the decline stage of the specimen.
4. During the gentle stage (C_i_D_i_), due to the buckling of the specimen, its vertical displacement increases rapidly. However, whether the specimen undergoes acid rain corrosion or not, it has a stable bearing capacity in the gentle stage, which proves that the failure mode of the specimen is plastic failure.

### 3.4. Bearing Capacity

**Figure 8:**
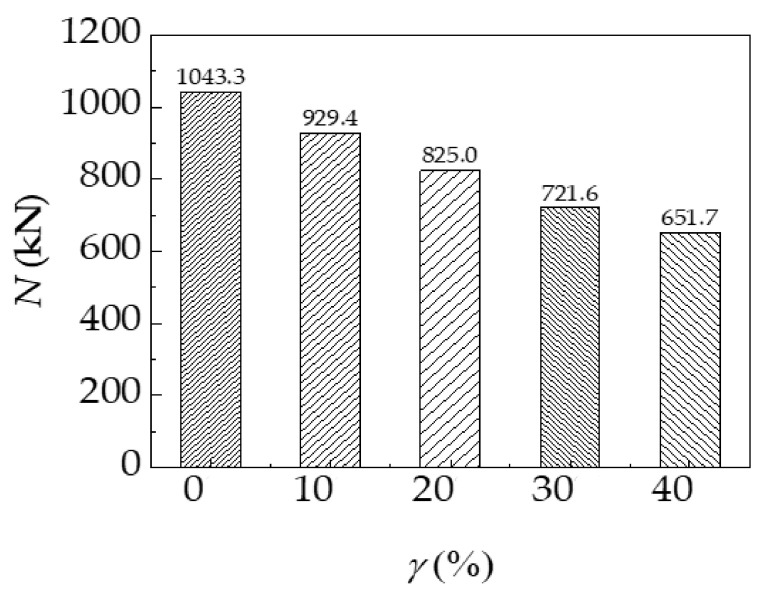
The ultimate bearing capacity of the specimen under different corrosion rates.

The ultimate bearing capacity of the specimen under different corrosion rates is shown in Figure 8. As the corrosion rate increases, the ultimate bearing capacity of the specimen decreases significantly. Compared with the specimen without corrosion, the ultimate bearing capacity of the specimen with 10, 20, 30, and 40% corrosion rates decreases by 10.9, 20.9, 30.8, and 37.6%, respectively. The results further demonstrate that acid rain has remarkable impacts on the ultimate bearing capacity of the CFST-LC.

## 4. Ultimate Bearing Capacity of the CFST-LC under Eccentric Compression

### 4.1. Parameter Analysis

Based on [1], a dimensionless parameter, corrosion damage factor β, is defined as Equation (28).

(28) $$\beta =\frac{\Delta t}{t}$$

where, *t* is the initial wall thickness of the steel tube, mm; ∆*t* is the wall thickness loss of the steel tube caused by corrosion, mm.

A parametric analysis is conducted to analyse the effects of section size (*B*), yield strength of steel tube (*f_y_*), axial compressive strength of concrete (*f_cu_*), steel ratio (*α*), slenderness ratio (*λ*), and load eccentricity (*e*) on the ultimate bearing capacity of the CFST-LC under eccentric compression. The default parameters of the specimen for simulation are:
*B* × *t* × *L* = 160 mm × 4 mm × 1200 mm*α* = 0.11*e* = 20 mm*f_cu_* = 40 MPa*f_y_* = 345 MPa


The corresponding ranges of those parameters are:
*B* = 200–500 mm*f_y_* = 235–420 MPa*f_cu_* = 30–60 MPa*α* = 0.05–0.23*λ* = 20–50*e* = 10–70 mm

**Figure 9:**
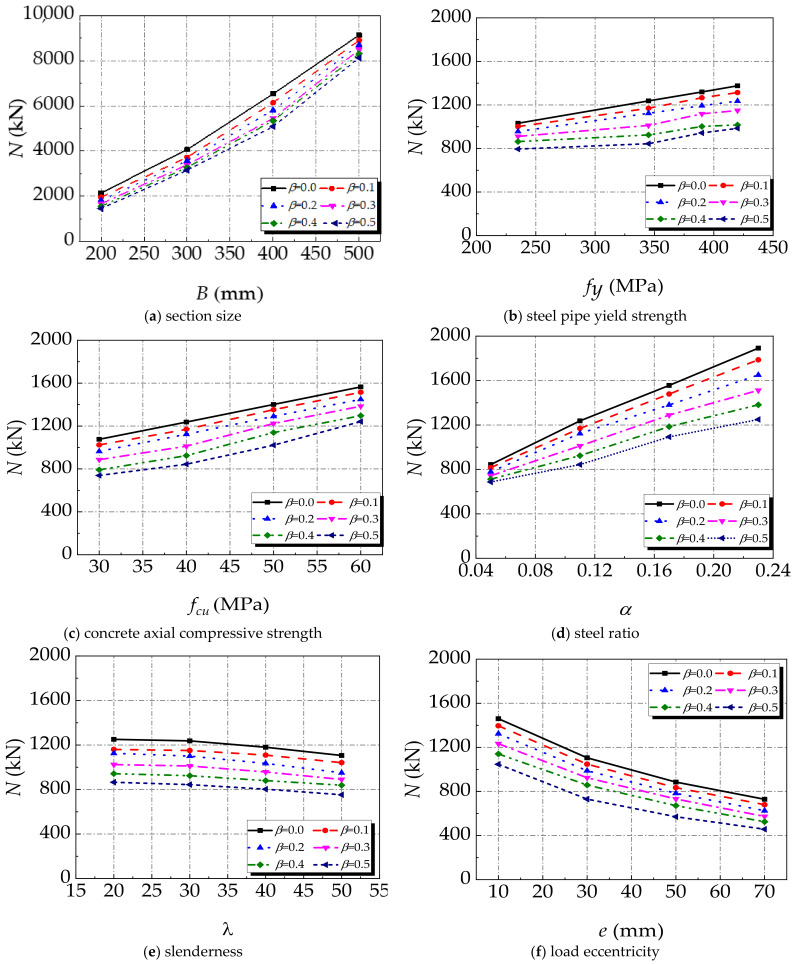
The influence of different parameters on the ultimate bearing capacity of specimens (**a**) section size; (**b**) steel pipe yield strength; (**c**) concrete axial compressive strength; (**d**) steel ratio; (**e**) slenderness; (**f**) load eccentricity.

The parametric analysis results are given in Figure 9. With the increasing of section size of the specimen, in Figure 9a, the rise tendency is different with different corrosion factor *β*. It indicates that the section size has a significant impact on the ultimate bearing capacity of the CFST-LC under eccentric compression. The increasement of the section size can largely enhance the ultimate bearing capacity of the specimen, for it amplifies the area of core concrete to improve the ultimate bearing capacity.

Figure 9 shows the influence of section size, yield strength of the steel tube, compressive strength of concrete, and steel ratio on the ultimate bearing capacity of the specimens. It can be seen that the ultimate bearing capacity of the specimen increases linearly with the increase in section size, yield strength of the steel tube, compressive strength of concrete and steel ratio. Although the increasement of the section size increases the core concrete area, the restraint effect of the steel tube on concrete decreases. When the section size becomes large enough, the specimen can be considered as very thin-walled members and the confinement effect has been wakened or has even vanished. Therefore, the steel tube and concrete are considered to bear axial load individually, as detailed in the superposition theory. After acid rain corrosion, the effective thickness of the steel tube decreases, but due to the increase in material strength, the confinement coefficient of the actual specimen increases, which improves the ultimate bearing capacity of the specimens. The ascending steel ratio increases the thickness of the steel tube, which leads to the improvement of steel tube bending stiffness. The buckling of steel is delayed, and the ultimate bearing capacity is improved.

Figure 9e,f depict the influence of slenderness ratio and load eccentricity on the ultimate load bearing capacity. It can be seen that slenderness ratio and load eccentricity greatly influence the ultimate bearing capacity of the specimen. With the increase in the slenderness ratio, the ultimate bearing capacity of the specimen gradually decreases, and the same tendency is suit for eccentricity. With the increase in slenderness ratio, the specimens are more likely to lose stability, which leads to instability failure. Therefore, in order to improve the ultimate bearing capacity of the specimen, the large slenderness ratio of the specimen should be avoided. The longitudinal bending deformation and the lateral deformation of the specimen increases after the CFST-LC is subjected to eccentric load, which makes the specimen prone to buckling failure. The buckling in the specimens decrease the ultimate bearing capacity. In the structural design, the excessive eccentric load should be avoided so that the ultimate bearing capacity of the component can be improved.

### 4.2. Design Method

The bearing capacity of a concrete-filled square steel tube member under axial compression at ambient temperature can be calculated by the following equations [36]:

(29) $$N_{0}=A_{sc}\times f_{sc}$$

(30) $$f_{sc}=(1.212+B\theta +C\theta^{2})f_{cu}$$

(31) $$\alpha_{sc}=\frac{A_{s}}{A_{c}}$$

(32) $$\theta =\frac{f_{y}}{f_{cu}}$$

(33) $$B=0.131f_{y}/213+0.723$$

(34) $$C=-0.07f_{cu}/14.4+0.026$$

in which, *N*_0_ is the compression bearing load of CFST-LC, kN; *f_sc_* design value of compressive strength of the concrete filled steel tube, MPa; *A_sc_* section area of concrete filled steel tubular members, mm^2^; *B*, *C* influence coefficient of section shape on hoop effect; *α_sc_* steel ratio of concrete filled steel tubular members; *θ* hoop coefficient of concrete filled steel tubular members; *f_y_* denotes the strength of the steel tube, N/mm^2^; *A_s_* is the cross-section area of the steel tube, mm^2^; *f_cu_* represents the strength of the concrete, in N/mm^2^; *A_c_* is the cross-section area of the concrete, mm^2^.

Additionally, reference [36] proposed the expressions for the bearing capacity of the CFST-LC beam-columns member under eccentric compression, without corrosion at ambient temperature, which are given by Equations (35)–(38). The eccentricity and the height of the compression zone of concrete are taken into consideration.

(35) $$\frac{N}{N_{0}}+(1-\alpha_{c})\frac{M}{M_{w}}=1$$

(36) $$M_{w}=\left[0.5A_{s}(B-2t-d_{n})+bt(t+d_{n})\right]f_{y}$$

(37) $$d_{n}=\frac{A_{S}-2Bt}{(b-2t)\frac{f_{c}}{f}+4t}$$

(38) M=Ne

where *M* is the bending moment of the column end; *α_c_* represents the concrete work bearing coefficient; *M_w_* gives the bending bearing capacity of the section where only the bending moment acts; *f_y_* is the bending strength of the steel; *B* denotes the side length of section; *t* is the wall thickness of the steel tube; *d_n_* is the height of compression zone of the concrete in the steel tube; *f* stands for the design value of the steel flexural strength.

When corrosion is taken into consideration, its impact on the properties of the material cannot be ignored. Therefore, the strength, cross-section area and wall thickness of the steel tube in Equations (29)–(38) should be corrected to Equations (39)–(41). By substituting Equations (39)–(41) into Equations (29)–(38), the expression of the bearing capacity of the SCFST-LC under eccentric compression after acid rain corrosion is obtained. The equations are given as follows:

(39) $$f_{ye}=(1-1.007\gamma )f_{y}$$

(40) $$A_{se}=4(B-2t+\gamma t)\gamma t$$

(41) $$t_{e}=(1-\gamma )t$$

**Figure 10:**
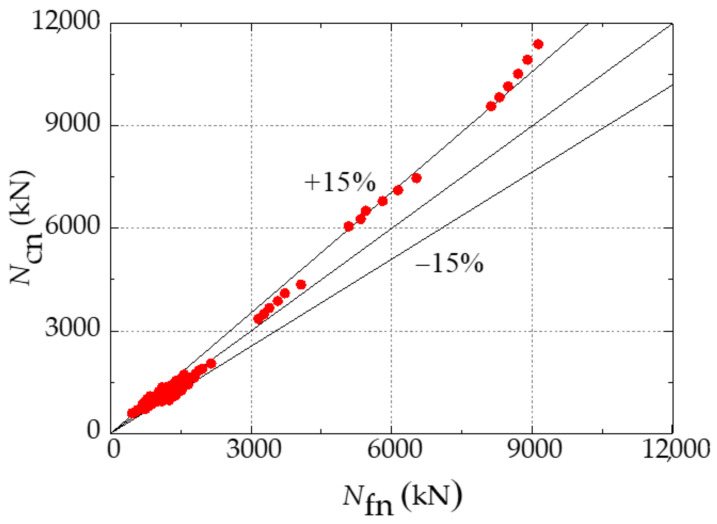
Comparison between the ultimate bearing capacity obtained by numerical modelling and simplified formula.

Numerical modelling is conducted to verify the accuracy of the attained theoretical equations. The numerical and theoretical results of the ultimate bearing capacity of 144 CFST-LC under eccentric compression after acid rain corrosion are attained and compared in Figure 10. There is no significant difference between the values of *N*_fn_ obtained by the finite element simulation and the simplified formula. The average and variance values of the ratio of *N*_fn_ to *N*_cn_ are 1.093 and 0.003, respectively. Although the theoretical results of a few specimens are slightly higher than those gained by numerical modelling, the error is within 15% and the calculation result is generally safe. Therefore, this simplified formula can accurately predict the bearing capacity of SCFST-LC under eccentric compression after acid rain corrosion. It can provide theoretical reference for safety and stability evaluation, as well as the prediction of service life of the CFST structure under the corroded environment.

## 5. Conclusions

In this paper, the corrosion damage of concrete-filled steel tubular (CFST) eccentrically loaded long columns after acid rain corrosion was simulated by the method of steel tube wall thickness and strength reduction. A simplified calculation formula for the bearing capacity of CFST eccentrically loaded long columns after acid rain corrosion was proposed. The finite element simulation is in good agreement with the formula calculation, which verifies the reliability of the formula.

1. The failure mode of the SCFST-LC under eccentric compression being subjected to acid rain corrosion was similar to that of the scenario without corrosion. Local buckling occurred at the mid-span part of the specimen and the internal concrete columns collapsed. The increase in the corrosion rate could amplify the local buckling amplitude at the mid-span part of the external steel tube.
2. The load-vertical displacement curves of the SCFST-LC under eccentric compression with different corrosion rates had a similar tendency, including elastic stage, elastic-plastic stage, descending stage, and gentle stage. The peak strain appeared earlier due to the increasing corrosion rate. Furthermore, the failure modes of both specimens, with or without corrosion, were plastic failure and they had a stable bearing capacity during the gentle stage.
3. The parametric analysis results demonstrated that the section size, yield strength of the steel tube, compressive strength of concrete, steel ratio, slenderness ratio, and load eccentricity all had significant effects on the ultimate bearing capacity of the SCFST-LC. The ultimate bearing capacity of the SCFST-LC was enhanced by the increase in its section size, material strength, and steel ratio. However, the growth of the slenderness ratio and load eccentricity could diminish its ultimate bearing capacity.
4. Through the parameter analysis of the results, the simplified formula for the ultimate bearing capacity of the SCFST-LC under eccentric compression after acid rain corrosion was proposed. Its accuracy and efficiency have been verified by comparing the bearing capacity obtained by the simplified formula and numerical modelling.

## Figures and Tables

**Table 1:** The main design parameters in [35].

Model Number	*B* × *L* × *t* (mm)	∆*t* (mm)	*f_cu_* (MPa)	*f_y_* (MPa)	*γ* (%)	*e* (mm)	*N_ue_* (kN)	Ref.
NC-50-0	160 × 1250 × 3.64	0	53.5	342.5	0	50	1095.00	[34]
NC-50-1	160 × 1250 × 3.64	0.36	53.5	342.5	10	50	970.00	
NC-50-2	160 × 1250 × 3.64	0.758	53.5	342.5	20	50	805.00	
NC-25-2	160 × 1250 × 3.64	0.758	53.5	342.5	20	25	1105.00	
S3-0-0	80 × 300 × 3.0	0.00	49.8	358	0	0	606.25	[35]
S3-0-10	80 × 300 × 3.0	0.30	49.8	358	10	0	543.75	
S3-0-20	80 × 300 × 3.0	0.60	49.8	358	20	0	493.75	
S3-0-30	80 × 300 × 3.0	0.90	49.8	358	30	0	456.25	
S4.5-0-0	80 × 300 × 4.5	0.00	49.8	358	0	0	774.78	
S4.5-0-10	80 × 300 × 4.5	0.45	49.8	358	10	0	704.35	
S4.5-0-20	80 × 300 × 4.5	0.90	49.8	358	20	0	610.44	
S4.5-0-30	80 × 300 × 4.5	1.35	49.8	358	30	0	547.83	

Note, *B* and *t* are the side length of specimen and the initial thickness of steel tube; ∆*t* is the wall thickness reduction of steel tube due to corrosion; *γ* is the corrosion rate; *N_ue_* is the test value of the bearing capacity of specimen; *e* is the load eccentricity.

**Table 2:** Numerical modelling results of specimens with acid rain corrosion.

Model Number	*B* × *L* × *t* (mm)	*N_ue_* (kN)	*N_ce_* (kN)	*N_be_* (kN)	*N_ue_*/*N_ce_*	*N_ue_*/*N_be_*	Ref.
NC-50-0	160 × 1250 × 3.64	1095	1078.48	1058.34	1.02	1.03	[34]
NC-50-1	160 × 1250 × 3.64	970	941.44	927.87	1.03	1.04	
NC-50-2	160 × 1250 × 3.64	805	840.82	825.35	0.96	0.98	
NC-25-2	160 × 1250 × 3.64	1105	1076.92	1133.61	1.03	0.97	
Average value					1.00	1.00	
Variance					0.001	0.001	
S3-0-0	80 × 300 × 3	606.25	-	625.46	-	0.97	[35]
S3-0-10	80 × 300 × 3	543.75	-	573.08	-	0.95	
S3-0-20	80 × 300 × 3	493.75	-	511.82	-	0.96	
S3-0-30	80 × 300 × 3	456.25	-	461.98	-	0.99	
S4.5-0-0	80 × 300 × 4.5	774.78	-	814.8	-	0.95	
S4.5-0-10	80 × 300 × 4.5	704.35	-	701.1	-	1.00	
S4.5-0-20	80 × 300 × 4.5	610.44	-	610.99	-	1.00	
S4.5-0-30	80 × 300 × 4.5	547.83	-	546.47	-	1.00	
Average value					-	0.98	
Variance					-	0.001	

Note, *N_ce_* is the numerical modeling results in references; *N_be_* is the numerical modeling results obtained by using the constitutive relation equation proposed in this paper.

**Table 3:** Main design parameters of models.

Model Number	*B* × *L* × *t* (mm)	∆*t* (mm)	*e* (mm)	*f_cu_* (MPa)	*f_y_* (MPa)	*γ* (%)	*α*	*N* (kN)
S-40-345-0.11-0	160 × 1200 × 4	0	40	40	345	0	0.11	1043.32
S-40-345-0.11-10	160 × 1200 × 4	0.4	40	40	345	10	0.11	929.41
S-40-345-0.11-20	160 × 1200 × 4	0.8	40	40	345	20	0.11	824.95
S-40-345-0.11-30	160 × 1200 × 4	1.2	40	40	345	30	0.11	721.63
S-40-345-0.11-40	160 × 1200 × 4	1.6	40	40	345	40	0.11	651.72

Note: In the model number, S represents that the cross-section of a specimen is square. The first number denotes the compressive strength of the concrete cube. The second number means the yield strength of the steel tube. The third number indicates the initial steel ratio of a specimen (*α* = *A_s_*/*A_c_*, *A_s_* is the initial cross-sectional area of the steel tube, *A_c_* represents the cross-sectional area of the concrete). The fourth number states the corrosion rate. For example, S-40-235-0.11-0 means that the specimen is a concrete-filled square steel tube column. The compressive strength of its concrete cube and the yield strength of its steel tube are 40 and 345 MPa, respectively. Its initial steel ratio is 0.11 and its corrosion rate is 0%.

## Data Availability

The data presented in this study are available on request from the corresponding author.
